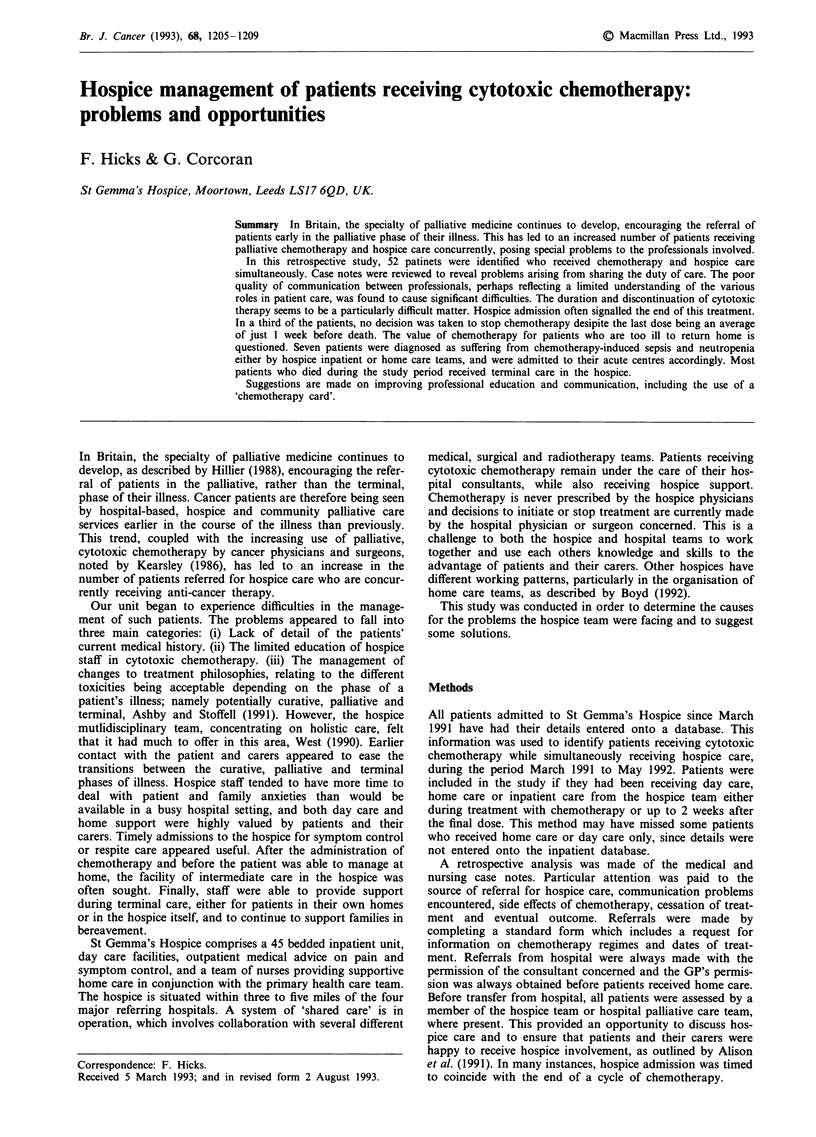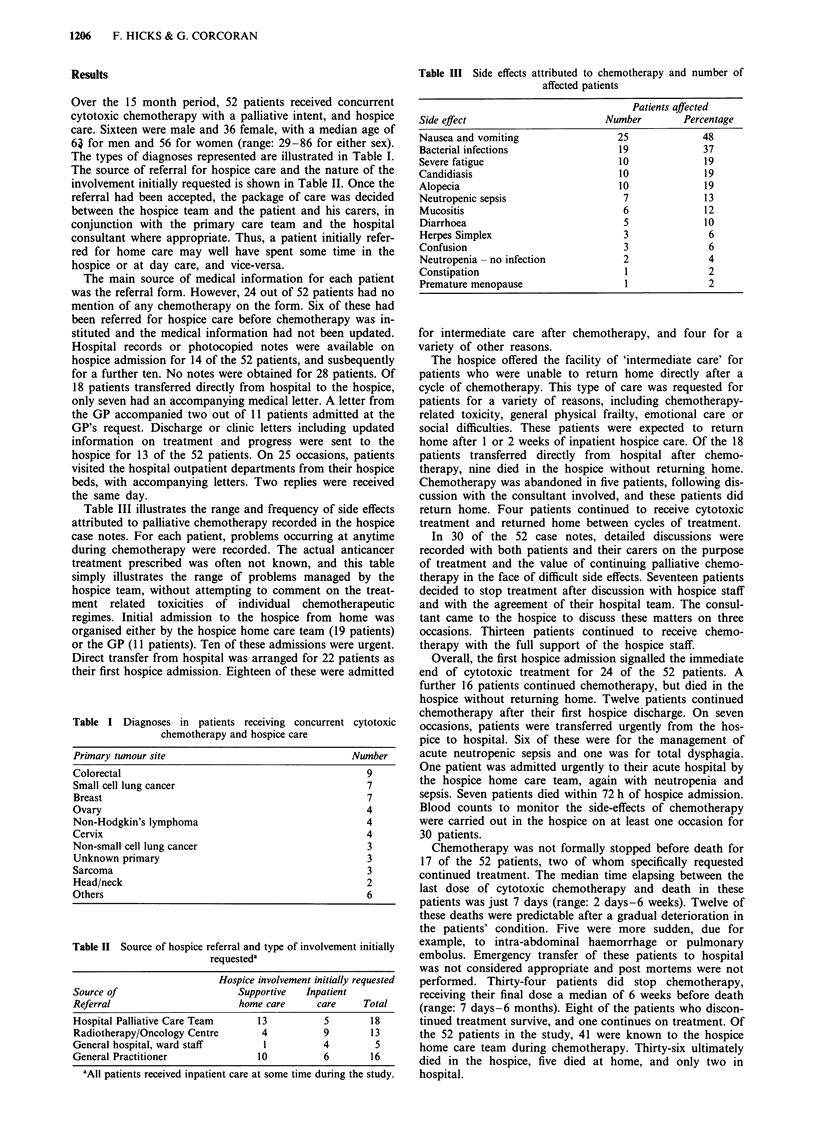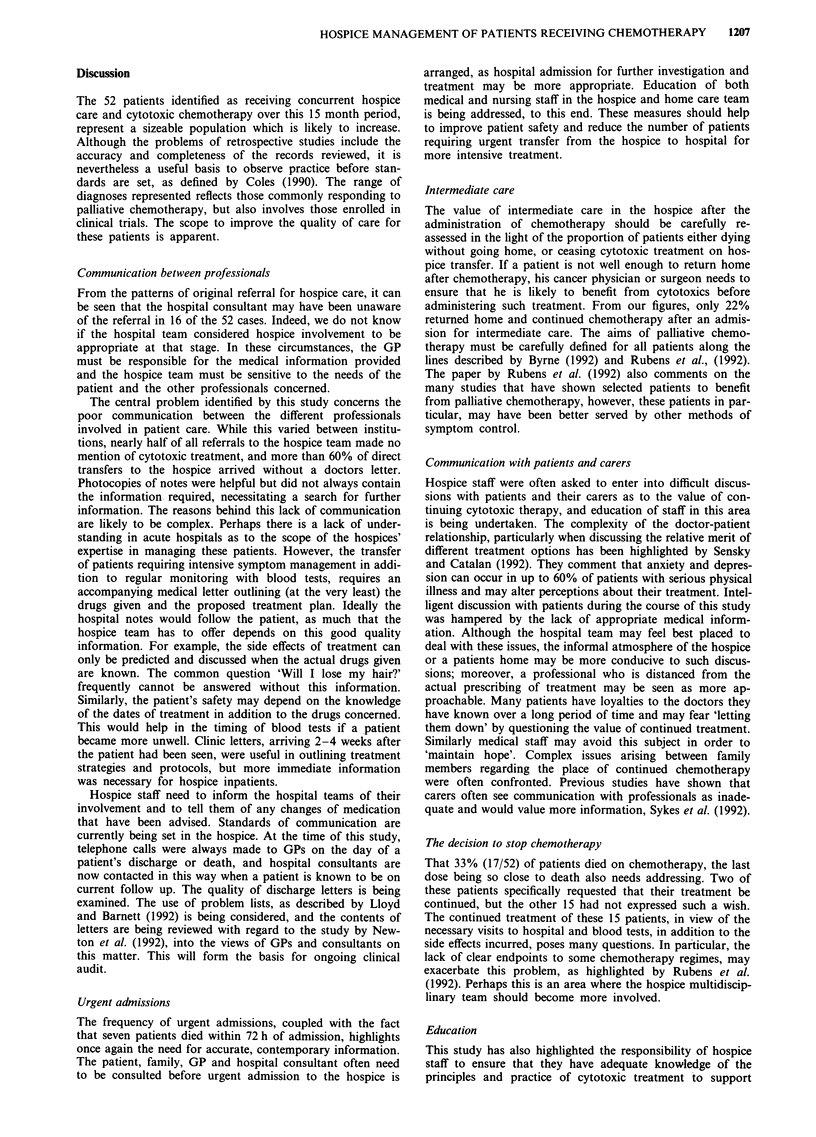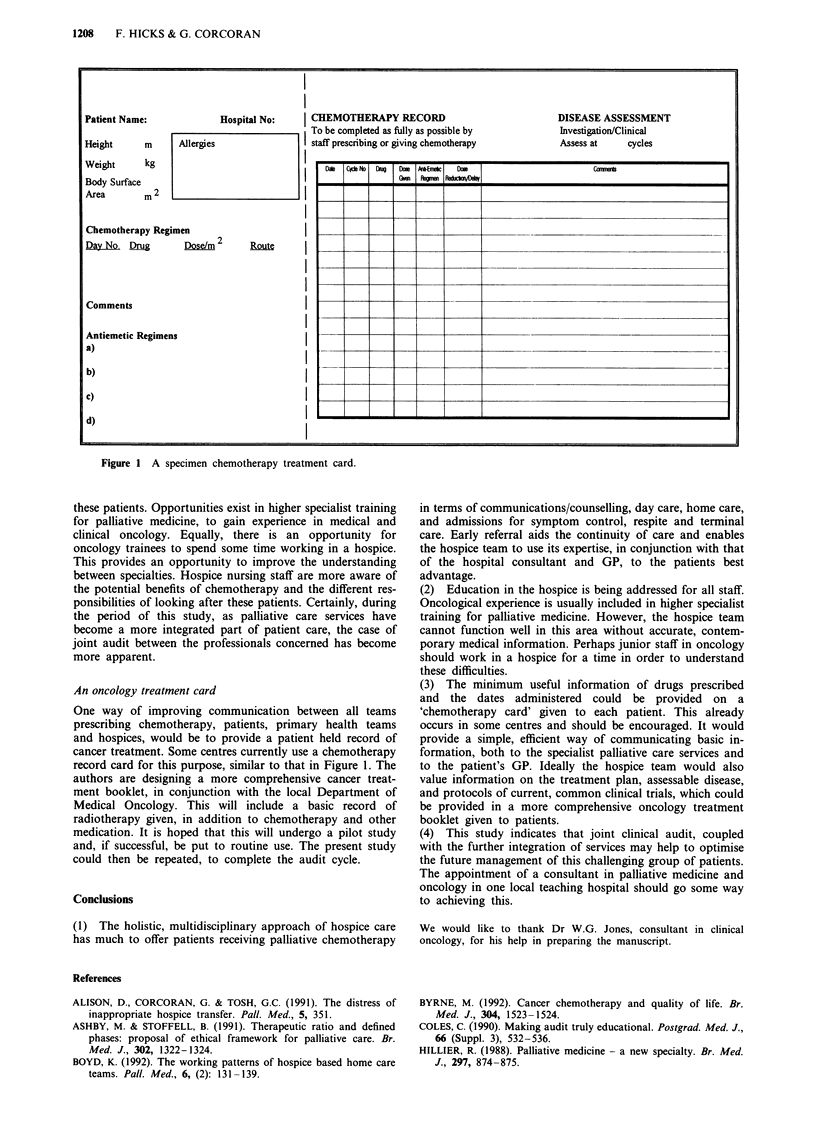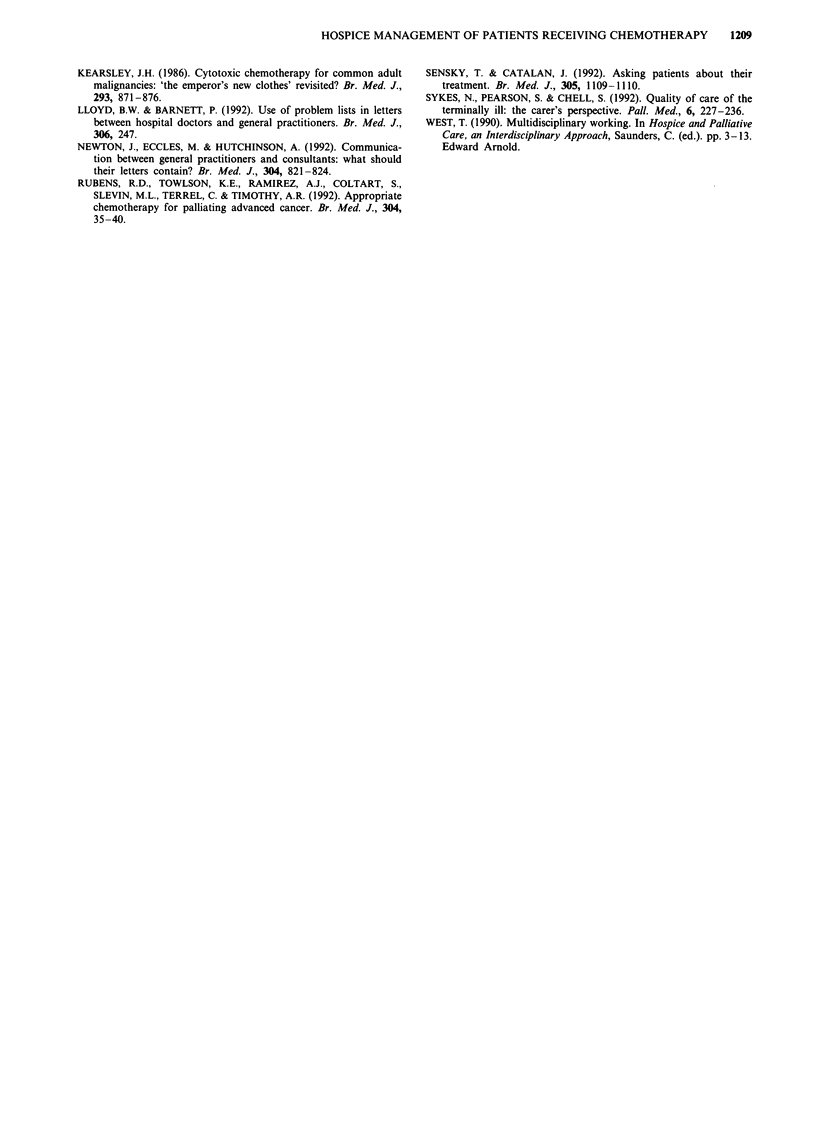# Hospice management of patients receiving cytotoxic chemotherapy: problems and opportunities.

**DOI:** 10.1038/bjc.1993.505

**Published:** 1993-12

**Authors:** F. Hicks, G. Corcoran

**Affiliations:** St Gemma's Hospice, Moortown, Leeds, UK.

## Abstract

In Britain, the specialty of palliative medicine continues to develop, encouraging the referral of patients early in the palliative phase of their illness. This had led to an increased number of patients receiving palliative chemotherapy and hospice care concurrently, posing special problems to the professionals involved. In this retrospective study, 52 patients were identified who received chemotherapy and hospice care simultaneously. Case notes were reviewed to reveal problems arising from sharing the duty of care. The poor quality of communication between professionals, perhaps reflecting a limited understanding of the various roles in patient care, we found to cause significant difficulties. The duration and discontinuation of cytotoxic therapy seems to be a particularly difficult matter. Hospice admission often signalled the end of this treatment. In a third of the patients, no decision was taken to stop chemotherapy despite the last dose being an average of just 1 week before death. The value of chemotherapy for patients who are too ill to return home is questioned. Seven patients were diagnosed as suffering from chemotherapy-induced sepsis and neutropenia either by hospice inpatient or home care teams, and were admitted to their acute centres accordingly. Most patients who died during the study period received terminal care in the hospice. Suggestions are made on improving professional education and communication, including the use of a 'chemotherapy card'.


Br. J. Cancer (1993), 68, 1205–1209 © Macmillan Press Ltd., 1993

### Hospice management of patients receiving cytotoxic chemotherapy: problems and opportunities

F. Hicks & G. Corcoran

*St Gemma's Hospice, Moortown, Leeds LS17 6QD, UK.*

**Summary** In Britain, the specialty of palliative medicine continues to develop, encouraging the referral of patients early in the palliative phase of their illness. This has led to an increased number of patients receiving palliative chemotherapy and hospice care concurrently, posing special problems to the professionals involved.

In this retrospective study, 52 patinets were identified who received chemotherapy and hospice care simultaneously. Case notes were reviewed to reveal problems arising from sharing the duty of care. The poor quality of communication between professionals, perhaps reflecting a limited understanding of the various roles in patient care, was found to cause significant difficulties. The duration and discontinuation of cytotoxic therapy seems to be a particularly difficult matter. Hospice admission often signalled the end of this treatment. In a third of the patients, no decision was taken to stop chemotherapy desipite the last dose being an average of just 1 week before death. The value of chemotherapy for patients who are too ill to return home is questioned. Seven patients were diagnosed as suffering from chemotherapy-induced sepsis and neutropenia either by hospice inpatient or home care teams, and were admitted to their acute centres accordingly. Most patients who died during the study period received terminal care in the hospice.

Suggestions are made on improving professional education and communication, including the use of a 'chemotherapy card'.

In Britain, the specialty of palliative medicine continues to develop, as described by Hillier (1988), encouraging the referral of patients in the palliative, rather than the terminal, phase of their illness. Cancer patients are therefore being seen by hospital-based, hospice and community palliative care services earlier in the course of the illness than previously. This trend, coupled with the increasing use of palliative, cytotoxic chemotherapy by cancer physicians and surgeons, noted by Kearsley (1986), has led to an increase in the number of patients referred for hospice care who are concurrently receiving anti-cancer therapy.

Our unit began to experience difficulties in the management of such patients. The problems appeared to fall into three main categories: (i) Lack of detail of the patients' current medical history. (ii) The limited education of hospice staff in cytotoxic chemotherapy. (iii) The management of changes to treatment philosophies, relating to the different toxicities being acceptable depending on the phase of a patient's illness; namely potentially curative, palliative and terminal, Ashby and Stoffell (1991). However, the hospice mutlidisciplinary team, concentrating on holistic care, felt that it had much to offer in this area, West (1990). Earlier contact with the patient and carers appeared to ease the transitions between the curative, palliative and terminal phases of illness. Hospice staff tended to have more time to deal with patient and family anxieties than would be available in a busy hospital setting, and both day care and home support were highly valued by patients and their carers. Timely admissions to the hospice for symptom control or respite care appeared useful. After the administration of chemotherapy and before the patient was able to manage at home, the facility of intermediate care in the hospice was often sought. Finally, staff were able to provide support during terminal care, either for patients in their own homes or in the hospice itself, and to continue to support families in bereavement.

St Gemma's Hospice comprises a 45 bedded inpatient unit, day care facilities, outpatient medical advice on pain and symptom control, and a team of nurses providing supportive home care in conjunction with the primary health care team. The hospice is situated within three to five miles of the four major referring hospitals. A system of 'shared care' is in operation, which involves collaboration with several different medical, surgical and radiotherapy teams. Patients receiving cytotoxic chemotherapy remain under the care of their hospital consultants, while also receiving hospice support. Chemotherapy is never prescribed by the hospice physicians and decisions to initiate or stop treatment are currently made by the hospital physician or surgeon concerned. This is a challenge to both the hospice and hospital teams to work together and use each others knowledge and skills to the advantage of patients and their carers. Other hospices have different working patterns, particularly in the organisation of home care teams, as described by Boyd (1992).

This study was conducted in order to determine the causes for the problems the hospice team were facing and to suggest some solutions.

### Methods

All patients admitted to St Gemma's Hospice since March 1991 have had their details entered onto a database. This information was used to identify patients receiving cytotoxic chemotherapy while simultaneously receiving hospice care, during the period March 1991 to May 1992. Patients were included in the study if they had been receiving day care, home care or inpatient care from the hospice team either during treatment with chemotherapy or up to 2 weeks after the final dose. This method may have missed some patients who received home care or day care only, since details were not entered onto the inpatient database.

A retrospective analysis was made of the medical and nursing case notes. Particular attention was paid to the source of referral for hospice care, communication problems encountered, side effects of chemotherapy, cessation of treatment and eventual outcome. Referrals were made by completing a standard form which includes a request for information on chemotherapy regimes and dates of treatment. Referrals from hospital were always made with the permission of the consultant concerned and the GP's permission was always obtained before patients received home care. Before transfer from hospital, all patients were assessed by a member of the hospice team or hospital palliative care team, where present. This provided an opportunity to discuss hospice care and to ensure that patients and their carers were happy to receive hospice involvement, as outlined by Alison *et al.* (1991). In many instances, hospice admission was timed to coincide with the end of a cycle of chemotherapy.

Correspondence: F. Hicks.
Received 5 March 1993; and in revised form 2 August 1993.

### Results

Over the 15 month period, 52 patients received concurrent cytotoxic chemotherapy with a palliative intent, and hospice care. Sixteen were male and 36 female, with a median age of 63 for men and 56 for women (range: 29–86 for either sex). The types of diagnoses represented are illustrated in Table I. The source of referral for hospice care and the nature of the involvement initially requested is shown in Table II. Once the referral had been accepted, the package of care was decided between the hospice team and the patient and his carers, in conjunction with the primary care team and the hospital consultant where appropriate. Thus, a patient initially referred for home care may well have spent some time in the hospice or at day care, and vice-versa.

The main source of medical information for each patient was the referral form. However, 24 out of 52 patients had no mention of any chemotherapy on the form. Six of these had been referred for hospice care before chemotherapy was instituted and the medical information had not been updated. Hospital records or photocopied notes were available on hospice admission for 14 of the 52 patients, and susbequently for a further ten. No notes were obtained for 28 patients. Of 18 patients transferred directly from hospital to the hospice, only seven had an accompanying medical letter. A letter from the GP accompanied two out of 11 patients admitted at the GP's request. Discharge or clinic letters including updated information on treatment and progress were sent to the hospice for 13 of the 52 patients. On 25 occasions, patients visited the hospital outpatient departments from their hospice beds, with accompanying letters. Two replies were received the same day.

Table III illustrates the range and frequency of side effects attributed to palliative chemotherapy recorded in the hospice case notes. For each patient, problems occurring at anytime during chemotherapy were recorded. The actual anticancer treatment prescribed was often not known, and this table simply illustrates the range of problems managed by the hospice team, without attempting to comment on the treatment related toxicities of individual chemotherapeutic regimes. Initial admission to the hospice from home was organised either by the hospice home care team (19 patients) or the GP (11 patients). Ten of these admissions were urgent. Direct transfer from hospital was arranged for 22 patients as their first hospice admission. Eighteen of these were admitted for intermediate care after chemotherapy, and four for a variety of other reasons.

The hospice offered the facility of 'intermediate care' for patients who were unable to return home directly after a cycle of chemotherapy. This type of care was requested for patients for a variety of reasons, including chemotherapy-related toxicity, general physical frailty, emotional care or social difficulties. These patients were expected to return home after 1 or 2 weeks of inpatient hospice care. Of the 18 patients transferred directly from hospital after chemotherapy, nine died in the hospice without returning home. Chemotherapy was abandoned in five patients, following discussion with the consultant involved, and these patients did return home. Four patients continued to receive cytotoxic treatment and returned home between cycles of treatment.

In 30 of the 52 case notes, detailed discussions were recorded with both patients and their carers on the purpose of treatment and the value of continuing palliative chemotherapy in the face of difficult side effects. Seventeen patients decided to stop treatment after discussion with hospice staff and with the agreement of their hospital team. The consultant came to the hospice to discuss these matters on three occasions. Thirteen patients continued to receive chemotherapy with the full support of the hospice staff.

Overall, the first hospice admission signalled the immediate end of cytotoxic treatment for 24 of the 52 patients. A further 16 patients continued chemotherapy, but died in the hospice without returning home. Twelve patients continued chemotherapy after their first hospice discharge. On seven occasions, patients were transferred urgently from the hospice to hospital. Six of these were for the management of acute neutropenic sepsis and one was for total dysphagia. One patient was admitted urgently to their acute hospital by the hospice home care team, again with neutropenia and sepsis. Seven patients died within 72 h of hospice admission. Blood counts to monitor the side-effects of chemotherapy were carried out in the hospice on at least one occasion for 30 patients.

Chemotherapy was not formally stopped before death for 17 of the 52 patients, two of whom specifically requested continued treatment. The median time elapsing between the last dose of cytotoxic chemotherapy and death in these patients was just 7 days (range: 2 days–6 weeks). Twelve of these deaths were predictable after a gradual deterioration in the patients' condition. Five were more sudden, due for example, to intra-abdominal haemorrhage or pulmonary embolus. Emergency transfer of these patients to hospital was not considered appropriate and post mortems were not performed. Thirty-four patients did stop chemotherapy, receiving their final dose a median of 6 weeks before death (range: 7 days–6 months). Eight of the patients who discontinued treatment survive, and one continues on treatment. Of the 52 patients in the study, 41 were known to the hospice home care team during chemotherapy. Thirty-six ultimately died in the hospice, five died at home, and only two in hospital.

**Table I** Diagnoses in patients receiving concurrent cytotoxic chemotherapy and hospice care

| *Primary tumour site* | *Number* |
|---|---|
| Colorectal | 9 |
| Small cell lung cancer | 7 |
| Breast | 7 |
| Ovary | 4 |
| Non-Hodgkin's lymphoma | 4 |
| Cervix | 4 |
| Non-small cell lung cancer | 3 |
| Unknown primary | 3 |
| Sarcoma | 3 |
| Head/neck | 2 |
| Others | 6 |

**Table II** Source of hospice referral and type of involvement initially requested[a]

| *Source of Referral* | *Hospice involvement initially requested* Supportive home care | Inpatient care | *Total* |
|---|---|---|---|
| Hospital Palliative Care Team | 13 | 5 | 18 |
| Radiotherapy/Oncology Centre | 4 | 9 | 13 |
| General hospital, ward staff | 1 | 4 | 5 |
| General Practitioner | 10 | 6 | 16 |

[a]All patients received inpatient care at some time during the study.

**Table III** Side effects attributed to chemotherapy and number of affected patients

| *Side effect* | *Patients affected* Number | Percentage |
|---|---|---|
| Nausea and vomiting | 25 | 48 |
| Bacterial infections | 19 | 37 |
| Severe fatigue | 10 | 19 |
| Candidiasis | 10 | 19 |
| Alopecia | 10 | 19 |
| Neutropenic sepsis | 7 | 13 |
| Mucositis | 6 | 12 |
| Diarrhoea | 5 | 10 |
| Herpes Simplex | 3 | 6 |
| Confusion | 3 | 6 |
| Neutropenia – no infection | 2 | 4 |
| Constipation | 1 | 2 |
| Premature menopause | 1 | 2 |

### Discussion

The 52 patients identified as receiving concurrent hospice care and cytotoxic chemotherapy over this 15 month period, represent a sizeable population which is likely to increase. Although the problems of retrospective studies include the accuracy and completeness of the records reviewed, it is nevertheless a useful basis to observe practice before standards are set, as defined by Coles (1990). The range of diagnoses represented reflects those commonly responding to palliative chemotherapy, but also involves those enrolled in clinical trials. The scope to improve the quality of care for these patients is apparent.

### *Communication between professionals*

From the patterns of original referral for hospice care, it can be seen that the hospital consultant may have been unaware of the referral in 16 of the 52 cases. Indeed, we do not know if the hospital team considered hospice involvement to be appropriate at that stage. In these circumstances, the GP must be responsible for the medical information provided and the hospice team must be sensitive to the needs of the patient and the other professionals concerned.

The central problem identified by this study concerns the poor communication between the different professionals involved in patient care. While this varied between institutions, nearly half of all referrals to the hospice team made no mention of cytotoxic treatment, and more than 60% of direct transfers to the hospice arrived without a doctors letter. Photocopies of notes were helpful but did not always contain the information required, necessitating a search for further information. The reasons behind this lack of communication are likely to be complex. Perhaps there is a lack of understanding in acute hospitals as to the scope of the hospices' expertise in managing these patients. However, the transfer of patients requiring intensive symptom management in addition to regular monitoring with blood tests, requires an accompanying medical letter outlining (at the very least) the drugs given and the proposed treatment plan. Ideally the hospital notes would follow the patient, as much that the hospice team has to offer depends on this good quality information. For example, the side effects of treatment can only be predicted and discussed when the actual drugs given are known. The common question 'Will I lose my hair?' frequently cannot be answered without this information. Similarly, the patient's safety may depend on the knowledge of the dates of treatment in addition to the drugs concerned. This would help in the timing of blood tests if a patient became more unwell. Clinic letters, arriving 2–4 weeks after the patient had been seen, were useful in outlining treatment strategies and protocols, but more immediate information was necessary for hospice inpatients.

Hospice staff need to inform the hospital teams of their involvement and to tell them of any changes of medication that have been advised. Standards of communication are currently being set in the hospice. At the time of this study, telephone calls were always made to GPs on the day of a patient's discharge or death, and hospital consultants are now contacted in this way when a patient is known to be on current follow up. The quality of discharge letters is being examined. The use of problem lists, as described by Lloyd and Barnett (1992) is being considered, and the contents of letters are being reviewed with regard to the study by Newton *et al.* (1992), into the views of GPs and consultants on this matter. This will form the basis for ongoing clinical audit.

### *Urgent admissions*

The frequency of urgent admissions, coupled with the fact that seven patients died within 72 h of admission, highlights once again the need for accurate, contemporary information. The patient, family, GP and hospital consultant often need to be consulted before urgent admission to the hospice is arranged, as hospital admission for further investigation and treatment may be more appropriate. Education of both medical and nursing staff in the hospice and home care team is being addressed, to this end. These measures should help to improve patient safety and reduce the number of patients requiring urgent transfer from the hospice to hospital for more intensive treatment.

### *Intermediate care*

The value of intermediate care in the hospice after the administration of chemotherapy should be carefully reassessed in the light of the proportion of patients either dying without going home, or ceasing cytotoxic treatment on hospice transfer. If a patient is not well enough to return home after chemotherapy, his cancer physician or surgeon needs to ensure that he is likely to benefit from cytotoxics before administering such treatment. From our figures, only 22% returned home and continued chemotherapy after an admission for intermediate care. The aims of palliative chemotherapy must be carefully defined for all patients along the lines described by Byrne (1992) and Rubens *et al.*, (1992). The paper by Rubens *et al.* (1992) also comments on the many studies that have shown selected patients to benefit from palliative chemotherapy, however, these patients in particular, may have been better served by other methods of symptom control.

### *Communication with patients and carers*

Hospice staff were often asked to enter into difficult discussions with patients and their carers as to the value of continuing cytotoxic therapy, and education of staff in this area is being undertaken. The complexity of the doctor-patient relationship, particularly when discussing the relative merit of different treatment options has been highlighted by Sensky and Catalan (1992). They comment that anxiety and depression can occur in up to 60% of patients with serious physical illness and may alter perceptions about their treatment. Intelligent discussion with patients during the course of this study was hampered by the lack of appropriate medical information. Although the hospital team may feel best placed to deal with these issues, the informal atmosphere of the hospice or a patients home may be more conducive to such discussions; moreover, a professional who is distanced from the actual prescribing of treatment may be seen as more approachable. Many patients have loyalties to the doctors they have known over a long period of time and may fear 'letting them down' by questioning the value of continued treatment. Similarly medical staff may avoid this subject in order to 'maintain hope'. Complex issues arising between family members regarding the place of continued chemotherapy were often confronted. Previous studies have shown that carers often see communication with professionals as inadequate and would value more information, Sykes *et al.* (1992).

### *The decision to stop chemotherapy*

That 33% (17/52) of patients died on chemotherapy, the last dose being so close to death also needs addressing. Two of these patients specifically requested that their treatment be continued, but the other 15 had not expressed such a wish. The continued treatment of these 15 patients, in view of the necessary visits to hospital and blood tests, in addition to the side effects incurred, poses many questions. In particular, the lack of clear endpoints to some chemotherapy regimes, may exacerbate this problem, as highlighted by Rubens *et al.* (1992). Perhaps this is an area where the hospice multidisciplinary team should become more involved.

### *Education*

This study has also highlighted the responsibility of hospice staff to ensure that they have adequate knowledge of the principles and practice of cytotoxic treatment to support

**Patient Name:** **Hospital No:**

Height m

Weight kg

Body Surface Area m$^2$

Allergies

**Chemotherapy Regimen**

Day No. Drug Dose/m$^2$ Route

**Comments**

**Antiemetic Regimens**

a)

b)

c)

d)

**CHEMOTHERAPY RECORD**

To be completed as fully as possible by staff prescribing or giving chemotherapy

**DISEASE ASSESSMENT**

Investigation/Clinical

Assess at cycles

| Date | Cycle No | Drug | Dose Given | Anti-Emetic Regimen | Dose Reduction/Delay | Comments |
|---|---|---|---|---|---|---|
| | | | | | | |
| | | | | | | |
| | | | | | | |
| | | | | | | |
| | | | | | | |
| | | | | | | |
| | | | | | | |
| | | | | | | |
| | | | | | | |
| | | | | | | |
| | | | | | | |
| | | | | | | |
| | | | | | | |
| | | | | | | |

**Figure 1** A specimen chemotherapy treatment card.

these patients. Opportunities exist in higher specialist training for palliative medicine, to gain experience in medical and clinical oncology. Equally, there is an opportunity for oncology trainees to spend some time working in a hospice. This provides an opportunity to improve the understanding between specialties. Hospice nursing staff are more aware of the potential benefits of chemotherapy and the different responsibilities of looking after these patients. Certainly, during the period of this study, as palliative care services have become a more integrated part of patient care, the case of joint audit between the professionals concerned has become more apparent.

### *An oncology treatment card*

One way of improving communication between all teams prescribing chemotherapy, patients, primary health teams and hospices, would be to provide a patient held record of cancer treatment. Some centres currently use a chemotherapy record card for this purpose, similar to that in Figure 1. The authors are designing a more comprehensive cancer treatment booklet, in conjunction with the local Department of Medical Oncology. This will include a basic record of radiotherapy given, in addition to chemotherapy and other medication. It is hoped that this will undergo a pilot study and, if successful, be put to routine use. The present study could then be repeated, to complete the audit cycle.

### Conclusions

(1) The holistic, multidisciplinary approach of hospice care has much to offer patients receiving palliative chemotherapy in terms of communications/counselling, day care, home care, and admissions for symptom control, respite and terminal care. Early referral aids the continuity of care and enables the hospice team to use its expertise, in conjunction with that of the hospital consultant and GP, to the patients best advantage.

(2) Education in the hospice is being addressed for all staff. Oncological experience is usually included in higher specialist training for palliative medicine. However, the hospice team cannot function well in this area without accurate, contemporary medical information. Perhaps junior staff in oncology should work in a hospice for a time in order to understand these difficulties.

(3) The minimum useful information of drugs prescribed and the dates administered could be provided on a 'chemotherapy card' given to each patient. This already occurs in some centres and should be encouraged. It would provide a simple, efficient way of communicating basic information, both to the specialist palliative care services and to the patient's GP. Ideally the hospice team would also value information on the treatment plan, assessable disease, and protocols of current, common clinical trials, which could be provided in a more comprehensive oncology treatment booklet given to patients.

(4) This study indicates that joint clinical audit, coupled with the further integration of services may help to optimise the future management of this challenging group of patients. The appointment of a consultant in palliative medicine and oncology in one local teaching hospital should go some way to achieving this.

We would like to thank Dr W.G. Jones, consultant in clinical oncology, for his help in preparing the manuscript.

### References

ALISON, D., CORCORAN, G. & TOSH, G.C. (1991). The distress of inappropriate hospice transfer. *Pall. Med.*, **5**, 351.

ASHBY, M. & STOFFELL, B. (1991). Therapeutic ratio and defined phases: proposal of ethical framework for palliative care. *Br. Med. J.*, **302**, 1322–1324.

BOYD, K. (1992). The working patterns of hospice based home care teams. *Pall. Med.*, **6**, (2): 131–139.

BYRNE, M. (1992). Cancer chemotherapy and quality of life. *Br. Med. J.*, **304**, 1523–1524.

COLES, C. (1990). Making audit truly educational. *Postgrad. Med. J.*, **66** (Suppl. 3), 532–536.

HILLIER, R. (1988). Palliative medicine – a new specialty. *Br. Med. J.*, **297**, 874–875.

KEARSLEY, J.H. (1986). Cytotoxic chemotherapy for common adult malignancies: 'the emperor's new clothes' revisited? *Br. Med. J.*, **293**, 871–876.

LLOYD, B.W. & BARNETT, P. (1992). Use of problem lists in letters between hospital doctors and general practitioners. *Br. Med. J.*, **306**, 247.

NEWTON, J., ECCLES, M. & HUTCHINSON, A. (1992). Communication between general practitioners and consultants: what should their letters contain? *Br. Med. J.*, **304**, 821–824.

RUBENS, R.D., TOWLSON, K.E., RAMIREZ, A.J., COLTART, S., SLEVIN, M.L., TERREL, C. & TIMOTHY, A.R. (1992). Appropriate chemotherapy for palliating advanced cancer. *Br. Med. J.*, **304**, 35–40.

SENSKY, T. & CATALAN, J. (1992). Asking patients about their treatment. *Br. Med. J.*, **305**, 1109–1110.

SYKES, N., PEARSON, S. & CHELL, S. (1992). Quality of care of the terminally ill: the carer's perspective. *Pall. Med.*, **6**, 227–236.

WEST, T. (1990). Multidisciplinary working. In *Hospice and Palliative Care, an Interdisciplinary Approach*, Saunders, C. (ed.). pp. 3–13. Edward Arnold.